# H_2_S as a Bridge Linking Inflammation, Oxidative Stress and Endothelial Biology: A Possible Defense in the Fight against SARS-CoV-2 Infection?

**DOI:** 10.3390/biomedicines9091107

**Published:** 2021-08-28

**Authors:** Francesca Gorini, Serena Del Turco, Laura Sabatino, Melania Gaggini, Cristina Vassalle

**Affiliations:** 1 Institute of Clinical Physiology, National Research Council, 56124 Pisa, Italy; laura.sabatino@ifc.cnr.it (L.S.); melania.gaggini@ifc.cnr.it (M.G.); 2 Fondazione CNR-Regione Toscana G. Monasterio, 56124 Pisa, Italy

**Keywords:** endothelium, hydrogen sulfide, inflammation, therapeutic target, SARS-CoV-2, COVID-19

## Abstract

The endothelium controls vascular homeostasis through a delicate balance between secretion of vasodilators and vasoconstrictors. The loss of physiological homeostasis leads to endothelial dysfunction, for which inflammatory events represent critical determinants. In this context, therapeutic approaches targeting inflammation-related vascular injury may help for the treatment of cardiovascular disease and a multitude of other conditions related to endothelium dysfunction, including COVID-19. In recent years, within the complexity of the inflammatory scenario related to loss of vessel integrity, hydrogen sulfide (H_2_S) has aroused great interest due to its importance in different signaling pathways at the endothelial level. In this review, we discuss the effects of H_2_S, a molecule which has been reported to demonstrate anti-inflammatory activity, in addition to many other biological functions related to endothelium and sulfur-drugs as new possible therapeutic options in diseases involving vascular pathobiology, such as in SARS-CoV-2 infection.

## 1. Introduction

The endothelium is the inner lining that covers all blood vessels with a very large spatial distribution, and consequently with potentially different characteristics depending on its position in the body. Not just an inert barrier, the endothelium is recognized as a biologically active tissue that regulates vascular tone and structure through autocrine, paracrine, and hormone-like mechanisms [1]. In fact, it may respond to various stimuli (e.g., shear stress on the endothelial surface) and release numerous vasoactive substances with vasodilator or vasoconstrictor properties [1]. Loss of physiological homeostasis leads to endothelial dysfunction, a condition underlying micro- and macrovascular diseases, characterized by reduced capacity for vasodilation, recruitment of neutrophils, enhanced inflammation and oxidative stress, prothrombotic properties, impaired cell growth and vessel permeability [1]. Inflammatory processes remain critical determinants which can provoke increases in prothrombotic and pro-oxidative events. Therefore, endothelial dysfunction has been observed associated with different clinical diseases, such as chronic kidney disease, liver failure, atherosclerosis, hypertension, dyslipidemia, diabetes, and obesity [2]. This implies that endothelial dysfunction can have important repercussions on health and on the onset and development of practically all diseases. In particular, endothelial dysfunction can be promoted or exacerbated by severe acute respiratory syndrome coronavirus-2 (SARS-CoV-2) infection, either by direct (interaction of SARS-CoV-2 virus with endothelial angiotensin-converting enzyme 2 (ACE2)) or indirect mechanisms (e.g., systemic inflammation, leukocyte recruitment, immune dysregulation, procoagulant state, impaired fibrinolysis, or activation of the complement system) [3,4,5,6]. Conversely, preexisting endothelial dysfunction underlies cardiovascular risk factors (e.g., hypertension, diabetes, obesity, and aging), all conditions which may favor the risk and severity of novel coronavirus disease 2019 (COVID-19) [4]. Increasing experimental, clinical, and translational findings suggest that many common drugs, such as lipid-lowering, antihypertensive, and antidiabetic drugs, and also antioxidants (e.g., vitamin C and E, N-acetylcysteine-NAC) and anti-inflammatories (e.g., cyclooxygenase-2 inhibitors, and glucocorticoids) may target the endothelium [2]. Consequently, further knowledge of endothelium pathophysiology may greatly help in patient care, given its enormous diagnostic and therapeutic potential.

In this context, the role of hydrogen sulfide (H_2_S), defined as the third endogenous gaseous signaling molecule besides nitric oxide (NO) and carbon monoxide (CO), in recent years has aroused a great interest due to its importance on different signaling pathways at the endothelial level [5]. In particular, given the close interaction between reduced H_2_S levels and endothelial dysfunction and inflammation, and the relationship between NO and H_2_S, the therapeutic release of H_2_S may represent a new and intriguing development in the prevention and treatment of inflammatory-related endothelial dysfunction conditions [6].

In this review, we discuss the effects of H_2_S and the use of sulfur-drugs as a new possible therapeutic option in diseases involving vascular pathobiology, such as in the SARS-CoV-2 infection.

## 2. Hydrogen Sulfide: An Additive Key Factor in Vascular Homeostasis

In the last few years, the role of H_2_S as an interesting novel mediator involved in inflammation and endothelial function has emerged [7], in terms of the relaxation of blood vessels, regulation of blood pressure, reduction of inflammatory response, and induction of antioxidant defense [8].

This gasotransmitter is mostly produced through the reverse trans-sulfuration pathway by three different enzymes. Two enzymes, cystathionine beta synthase (CBS) and cystathionine gamma lyase (CSE), use piridoxal 5′ phosphate (PLP) as a cofactor, while 3-mercaptopiruvate sulfur transferase (3-MST), primarily located in the mitochondria, is not dependent on PLP [9,10].

**Figure 1:**
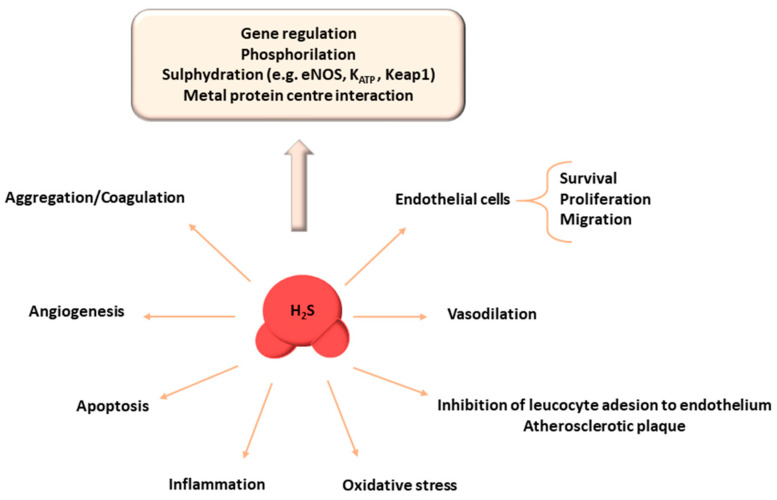
Main molecular, cellular, and systemic actions of hydrogen sulfide. H_2_S attenuates apoptosis and decreases oxidative stress by a direct action, upregulating the cellular antioxidant system. H_2_S also produces an anti-inflammatory response through the inhibition or induction of specific pathways. At the vascular level, in addition to promoting cell proliferation and migration, it induces angiogenesis, vasodilation, represses aggregation and coagulation, and reduces aortic atherosclerotic plaque formation. The mechanisms underlying the effects of H_2_S include the phosphorylation and addition of cysteine residues to target proteins, interaction with the metal protein centers of proteins, and gene regulation (for more details see text). Abbreviations: eNOS: Endothelial nitric oxide synthase; H_2_S: Hydrogen sulfide; K_ATP_: ATP-sensitive potassium channels; Keap1: Kelch-like ECH-associating protein 1.

The protective effect of H_2_S is mediated by many different cellular and molecular mechanisms (Figure 1).

S-sulfhydration, a chemical modification on specific cysteine residues of target proteins to form a persulfide group (–SSH), is considered a primary mechanism through which H_2_S alters the function of signaling proteins [11,12]. A striking example is the S-sulfhydration of endothelial nitric oxide synthase (eNOS), which promotes eNOS dimer stability, NO production, and consequent vasorelaxation [13]. Indeed, sulfhydration of the Kir 6.1 subunit of ATP-sensitive potassium (K_ATP_) channels activates the channel, causing vascular endothelial and smooth muscle cell hyperpolarization and vasorelaxation [14]. Accordingly, exogenous administration of H_2_S attenuates the rise of blood pressure in both spontaneously hypertensive rats [15] and in mice rendered hypertensive with angiotensin II (AngII) [16].

Although H_2_S may directly inactivate reactive oxygen species (ROS), e.g., through the inhibition of peroxynitrite-mediated processes in vivo [17], it also protects cells via the upregulation of antioxidant defense systems [6,8]. Specifically, sodium hydrosulfide (NaHS, an H_2_S donor) induces the sulfhydration of Kelch-like ECH-associating protein 1 (Keap1), a repressor of nuclear-factor-E2-related factor-2 (Nrf2), which is the main regulator of the antioxidant response. This action results in Keap1/Nrf2 disassociation, Nrf2 nuclear translocation, and increased mRNA expression of Nrf2-targeted downstream genes [18,19]. In human umbilical vein endothelial cells (HUVECs) exposed to H_2_O_2_, H_2_S upregulates a wide range of enzymes attenuating oxidative stress, such as catalase (CAT), superoxide dismutase (SOD), glutathione peroxidase, and glutathione-S-transferase [20]. Moreover, in endothelial cells (ECs) and fibroblasts, H_2_S-induced S-sulfhydration and activation of mitogen-activated extracellular signal-regulated kinase 1 are followed by phosphorylated ERK1/2 translocation into the nucleus to stimulate activity of PARP-1, a nuclear protein that exerts an important role in DNA damage repair [21]. H_2_S is further implicated in the regulation of the other major cellular inflammatory signaling pathway, namely the nuclear factor-κB (NF-κB) pathway [22]. The multifunctional pro-inflammatory cytokine, tumor necrosis factor α (TNF-α), stimulates the transcription of CSE. Consequently, the H_2_S-induced sulfhydration of the p65 subunit of NF-κB, promotes transcription of anti-apoptotic genes by enhancing its ability to bind the co-activator ribosomal protein S3 [23]. On the other hand, NaHS can exert an anti-inflammatory effect in ECs by inhibiting the expression of adhesion molecules (i.e., intercellular adhesion molecule-1 (ICAM-1), vascular cell adhesion molecule-1, P-selectin, and E-selectin), an early marker of endothelial activation and dysfunction, by upregulating the cytoprotective enzyme heme oxygenase-1 (HO-1), and decreasing TNF-α-induced NF-kB activation and ROS production [24]. Exogenous H_2_S also attenuates the inflammation and cytotoxicity induced by AngII in HUVECs via inhibition of the NF-κB/endothelin-I signaling pathway [25]. The pretreatment of HUVECs with NaHS can inhibit high-glucose-induced ICAM-1 expression at both the protein and mRNA levels, leading to a reduction of ROS production and NF-kB activity [26]. Furthermore, the administration of NaHS to high-glucose-treated ECs attenuates both the apoptosis and impairment of the CAT and SOD expression and activities induced by type 2 diabetes [27]. Interestingly, both diabetic rats [27] and patients with type 2 diabetes display markedly decreased plasma H_2_S levels [28,29].

H_2_S has also been ascertained to interact with the metal centers of proteins [30]. The interaction of H_2_S with the oxygenated form of human hemoglobin and myoglobin produces a sulfheme protein complex that participates in the catabolism of H_2_S [31]. Of interest is that H_2_S affects the function of soluble guanylyl cyclase (sGC), a receptor for NO, through the reduction of the ferric sGC heme into a ferrous state, facilitating NO-dependent vasodilation and thus providing an additional level of cross-talk between NO and H_2_S [32].

H_2_S stimulates EC proliferation, adhesion and migration in vitro and in vivo [6,11]. The mechanism whereby H_2_S regulates the key steps of angiogenesis can be driven by the increase in intracellular calcium levels in vascular ECs through the activation of many calcium-dependent signaling pathways and enzymes [9,33,34]. Also, H_2_S donors activate the PI-3K/Akt axis and enhance the phosphorylation of components of the MAPK pathway (p38 and ERK1/2) in EC models in vitro, with a subsequent pro-angiogenic effect [35]. Other mechanisms in H_2_S-induced angiogenesis in vascular ECs could also involve the opening of K_ATP_ channels by persulfidation of sulfonylurea receptor 1 subunit [36] and the activation of vascular endothelial growth factor receptor 2 (VEGFR2), a receptor tyrosine kinase that mediates most of the biological effects of the vascular endothelial growth factor through the breakage of a cysteine-cysteine disulfide bond within VEGFR2 [37].

Importantly, altered H_2_S metabolism is likely to be involved in the initiation and progression of atherosclerosis [9]. The H_2_S donors NaHS and GYY4137 may decrease aortic atherosclerotic plaque formation, macrophage infiltration, and aortic inflammation, and may partially restore endothelium-dependent relaxation in the aorta of apolipoprotein E (ApoE) gene-knockout mice [38,39]. In addition, CSE/H_2_S treatment directly sulfhydrated sirtuin-1, a histone deacetylase with a crucial role in longevity, increasing its activity and stability, thereby reducing atherosclerotic plaque formation in the aorta of these animals [39].

A few studies finally have investigated the anti-aggregatory and anticoagulatory effects of H_2_S [40]. In mice, GYY4137 seems to act as an anti-thrombotic and to regulate thrombogenesis by reducing platelet activation and adhesion molecule-mediated aggregation [41]. In mice treated with the H_2_S donor sodium sulfide (Na_2_S), thrombus formation induced using a phototoxic light/dye-injury model is significantly delayed compared to controls, due to the up-regulation of eNOS and inducible NOS (iNOS) [40]. Likewise, in an in vitro study on human whole blood, GYY4137 was observed to reduce platelet-leukocyte aggregation provoked by the thrombin-receptor activating peptide, and consequently facilitate microvascular thrombolysis [42].

It is important to note that H_2_S, in biological systems, co-exists with the sulfane sulfur species, i.e., uncharged sulfur atoms carrying six valence electrons being able to attach reversibly to other sulfur atoms [43]. Sulfane sulfur does not exist in the free form and can be considered as a sort of H_2_S storage, releasing H_2_S under reducing conditions, following a physiological signal [44,45]. Inorganic hydrogen polysulfides (H_2_S_n_, *n* ≥ 2), in particular, are endogenously produced through several enzymatic routes involving 3-MST [46], copper/zinc SOD [47], or the direct reaction between H_2_S and NO [48]. H_2_S_n_ is greatly reactive, and has recently emerged as a potential signaling molecule that immediately reacts with intracellular cysteine, glutathione (GSH), and protein cysteine residues [49].

### 2.1. Sulfur-Drugs as New Therapeutic Options in Endothelial Dysfunction

The use of sulfur moieties as therapeutic agents in a wide array of applications (e.g., arterial hypertension, atherosclerosis, myocardial hypertrophy, heart failure, ischemia-reperfusion, diabetic nephron- and retinopathy, and chronic inflammatory diseases), has attracted growing attention in the last few years [50,51] (Table 1).

#### 2.1.1. H_2_S Donors

Inorganic sulfide salts such as NaHS and Na_2_S are cheap and readily available, and have been largely employed in vitro and in animal models with the main effects of protecting ECs from inflammation, oxidative stress, damage induced by hyperglycemia, and promoting vasorelaxation and neovascularization, as illustrated above [6,52]. Nevertheless, they are unsuitable for clinical use, owing to the fast increase in H_2_S concentration to supraphysiological concentration, the change of intracellular pH if used in unbuffered solution, and possible toxicity characterized by pro-inflammatory effects [50,53]. In fact, in experimental studies, sulfide salts are frequently used at a very high concentration (100 μM to 10 mM), in great excess of the levels of H_2_S measured in vivo [52].

Unlike sulfide salts, the phosphorodithioate GYY4137 belongs to the class of organic slow-release H_2_S compounds, being able to release H_2_S over 3–4 h after dissolving [50]. GYY4137 has been demonstrated to exert vasodilator, antihypertensive, anti-atherosclerotic, and anti-thrombotic activities [38,41]. Whereas NaHS increases the synthesis of the interleukins (IL)-1β and -6, NO and prostaglandin E2 at a high concentration, GYY4137 inhibits the release of these pro-inflammatory mediators in a dose-dependent manner and also promotes the production of the anti-inflammatory chemokine IL-10 in lipopolysaccharide (LPS)-treated macrophages [53].

Diphosphorothioates such as AP67 and AP105 derive from structural modifications of the phosphorodithioate core and, if compared to GYY4137, have the advantages of being able to be employed at a lower concentration, showing an even enhanced activity [52]. The novel mitochondria-targeted AP39 and AP123 were found to reduce the high-glucose-induced hyperpolarization of the mitochondrial membrane and inhibit ROS production in microvascular ECs, with a long-lasting effect suggesting their application in the treatment of diabetic vascular complications [54]. The H_2_S prodrug sodium polysulthionate (SG1002) has been successfully used in both a swine model of acute limb ischemia [55] and in heart failure patients [56], in which it promotes an increase in circulating H_2_S and NO and, consequently, coronary artery vasorelaxation.

Garlic, which has been associated with multiple health beneficial effects in folk medicine for centuries, is rich in organosulfur compounds considered responsible for most of its pharmacological activities [57]. In particular, garlic-derived organic polysulfides like diallyl disulfide and diallyl trisulfide, as well as their analogs, act as H_2_S donors in the presence of GSH, and promote vasorelaxation (NO bioavailability) [57], lowering of arterial blood pressure [58], decreasing apoptosis and oxidative stress [59], and improved angiogenesis [60]. Similarly, the natural isothiocyanates commonly present in the Brassicaceae (e.g., broccoli, mustard, horseradish, rocket salad), can be considered as potential slow and long-lasting H_2_S donors able to release H_2_S in cell environments with high concentration of GSH and cysteine [61,62]. Sulforaphane and other isothiocyanates (i.e., benzyl isothiocyanate and phenethyl isocyanate) have anti-inflammatory properties mediated through the upregulation of HO-1 [63,64] and the glutamine cysteine ligase that plays a critical role in maintaining GSH homeostasis [63]. A diet based on sulforaphane-enriched foods was reported to significantly reduce oxidative stress related to improved endothelial-dependent relaxation of the aorta and lower blood pressure in spontaneously hypertensive rats [65].

Although L-cysteine is frequently employed in experimental studies to increase the production of endogenous H_2_S, it is not suitable for clinical use due to its unstable nature, being metabolized in a number of pathways including GSH synthesis, taurine synthesis and oxidation to sulfate [51]. Conversely, NAC, a well-tolerated compound used in clinical settings to enhance cellular levels of GSH, could represent a promising compound able to generate H_2_S [6], since the administration of NAC prevents the development of hypertension in rodents [66] and humans (Clinical Trial NCT01232257, 2011). Supplementation of taurine (2-aminoethanesulfonic acid), a metabolite of cysteine, has the ability to moderately lower blood pressure in prehypertensive subjects via endothelium-dependent and endothelium-independent vasodilation [67,68]. Like NAC, these antihypertensive effects are associated with increased expression of CSE and CBS, as observed both in the aorta of spontaneously hypertensive rats and in human vascular tissue cultures [67].

Synthetic cysteine derivatives such as S-propyl-cysteine, S-allyl-cysteine, and S-propargyl-cysteine (SPRC) are also enzymatically converted to H_2_S by CSE and CBS [50]. SPRC, though not available for clinical use [68], can attenuate the LPS-induced inflammatory response in cardiac myocytes by reducing the mRNA expression of TNF-α, ICAM-1 and iNOS [69], increase H_2_S levels through the upregulation of CSE, and protect HUVECs from TNF-α-induced dysfunction [70].

#### 2.1.2. H_2_S-Hybrid Drugs

In addition to H_2_S donors, there is a group of compounds known as H_2_S-hybrid drugs that, while able to release H_2_S, have a mechanism of action independent of H_2_S-mediated properties [61]. The group of ACE inhibitors represents one of the fundamental drug classes for the antihypertensive treatment [52]. Many of them, e.g., Omaprilat, Remikiren, Macitentan, Bosentan, Vardenafil, Sildenafil, have a sulfonil moiety in their structure [51]. Captocapril contains a thiol that, in plasma, can react with other thiol-containing compounds (cysteine, GSH) to form mixed disulfides, however it remains to be clarified whether the drug’s effects depend on sulfide signaling [6,51]. On the other hand, the pro-angiogenic [71], anti-inflammatory [72], and anti-apoptotic [73] activities of Zofenopril in ECs are at least partially mediated by its ability to increase H_2_S availability [72]. Of interest, Zofenopril, but not Enalapril (a non-thiol ACE inhibitor), also improves the vascular response to acetylcholine in spontaneously hypertensive rats, which is accompanied by increased H_2_S concentration in the plasma and the vascular wall [74].

Non-steroidal anti-inflammatory drugs (NSAIDs) are among the most widely used classes of medicines [75]. H_2_S-releasing derivatives of NSAIDs, synthetized by the conjugation of the parent NSAID with a dithiolethione moiety, show improved efficacy and reduced toxicity (i.e., gastrolesivity), which are mainly attributable to intracellular H_2_S/GSH formation in comparison to the non-releasing H_2_S compounds [75,76,77]. In particular, S-aspirin (ACS14), which exerts anti-platelet [78] and antithrombotic [79] activity in vivo and in vitro, prevents the formation and development of atherosclerosis in ApoE-deficient mice [80] and attenuates the oxidative stress caused by methylglyoxal (a chemically active metabolite of glucose and fructose) and high glucose in vascular smooth cultured cells, indicating a possible future use in the treatment of diabetic patients [81]. Likewise, S-diclofenac (ACS15) produces marked anti-inflammatory effects but significantly less gastric toxicity than diclofenac [82]. Notably, this drug can inhibit the smooth muscle cell growth that has been recognized as a fundamental event in vascular injury in diseases such as atherosclerosis [83].

### 2.2. H_2_S-Producing Compounds: A Further Tool against COVID-19

**Figure 2:**
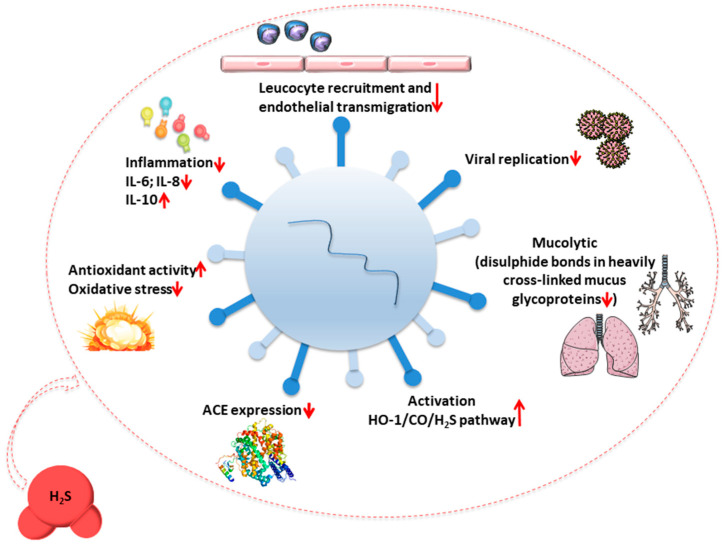
Primary biological mechanisms involving H_2_S (donors) in the protective response against SARS-CoV-2. Following viral infection, H_2_S counteracts inflammation by decreasing levels of IL-6 and IL-8, inducing an increase in IL-10 concentration and inhibiting the recruitment of leukocytes to the endothelium. It also represses ACE expression, which in turn leads to vasodilation, decreased inflammation and oxidative stress, and reduced fibrosis. H_2_S donors enhance endogenous antioxidant defenses and play a mucolytic role. The activity of H_2_S also results in the activation of the HO-1/CO/H_2_S system and inhibition of virus replication. Abbreviations: ACE: Angiotensin-converting enzyme; CO: Carbon monoxide; H_2_S: Hydrogen sulfide; HO-1: Heme oxygenase-1; IL: Interleukin.

A growing body of evidence supports the potential role of H_2_S as an effective host defense factor against SARS-CoV-2 [84,85] (Figure 2).

In a SARS-CoV-2 infection, a pro-inflammatory cytokine storm is a primary event characterized by increases in IL-1β, IL-6, and TNF-α [85], and even moderately elevated IL-6 levels have been associated with a high risk of respiratory failure in COVID-19 patients [86]. Interestingly, the 4-day change in ratio of IL-6 to IL-10 (a cytokine with anti-inflammatory effects involved both in innate and adaptive immunity), named the Dublin–Boston score, has proven to be a more reliable tool than IL-6 alone in predicting clinical progression and poor outcome in COVID-19 patients [87,88].

H_2_S can significantly downregulate the IL-6/STAT3 signaling pathway that is implicated in inflammatory responses and cell apoptosis [89]. Of note, serum H_2_S levels were found to inversely correlate with IL-6 as well as with the severity and final outcome of pneumonia in a cohort of patients with COVID-19, suggesting that the reduction of H_2_S bioavailability may be considered a biomarker of enhanced pro-inflammatory response, whereas exogenous administration of H_2_S could represent a valuable strategy to counteract severe manifestations of the infection [90,91]. In addition to decreasing IL-6 and IL-8 levels and the infiltration of polymorphonuclear cells, NaHS increases plasma levels of IL-10 in an animal model of induced acute lung injury [92], and administering IL-10 to IL-10 deficient mice in turn restores H_2_S production and homocysteine metabolism [93]. H_2_S has also been reported to play a role in enhancing T cell activation [94] and, among the variety of cell types capable to produce IL-10, IL-10 generation by CD4(+) T cells is crucial to preventing early mortality caused by excessive inflammation [87].

The anti-inflammatory effects of H_2_S further encompass the regulation of the ACE/ACE2 balance, although the underlying mechanisms remain elusive [95]. Specifically, NaHS may promote, dose-dependently, the expression of ACE2, a key enzymatic component of the renin-angiotensin-aldosterone system that is recognized to have vasodilating, anti-inflammatory, antioxidant, and antifibrotic effects, by catalyzing the generation of Ang (1–7) from AngII in ECs [96,97]. Conversely, NaHS treatment reduces ACE expression in spontaneously hypertensive rats [98], consistent with the recognized enhanced anti-hypertensive role of ACE-inhibitors containing a sulfur moiety in their chemical structure (see the previous section).

The role of H_2_S in acute and chronic inflammatory pulmonary diseases has been extensively investigated [91]. Besides anti-inflammatory activities (e.g., reduced mRNA expression of NF-kB, macrophage inflammatory protein-2, and interferon regulatory factor 3 [99,100]), H_2_S donors increase endogenous antioxidant defenses (e.g., SOD, glutathione peroxidase, and glutathione reductase) and inhibit leukocyte recruitment and transmigration through the inflamed endothelium [100,101]. Notably, there is evidence indicating that higher levels of GSH may improve an individual’s responsiveness to viral infections, protecting host cells from oxidative damage of the lung [102]. De Flora et al. observed that a 6-month preventive administration of NAC, a known precursor of GSH, provides a significant attenuation of influenza and influenza-like episodes, especially in the elderly [103]. The addition of NAC to a conventional therapy ameliorates oxidative stress and inflammation parameters (i.e., a decrease of malondialdehyde and TNF-α, and an increase of total antioxidant capacity) in patients with pneumonia [104]. NAC can act both as a direct scavenger of free radicals [105] and as a mucolytic agent capable of reducing disulphide bonds in heavily cross-linked mucus glycoproteins [106]. Moreover, the efficacy of NAC in the treatment of patients with chronic bronchitis and chronic obstructive pulmonary disease has been documented in several clinical trials and meta-analyses [107].

The levels of GSH are also negatively related with COVID-19 severity, and patients with moderate and severe illnesses show increased levels of ROS and a higher ROS/GSH ratio than subjects with mild symptoms [102]. Hence, it is sensible to hypothesize that replenishing intracellular GSH could be a useful strategy against SARS-CoV-2, as shown in two cases in which a glutathione-based therapy (GSH and NAC) combined with antioxidants (alpha-lipoic acid and vitamin C) was immediately effective in relieving symptoms of dyspnea [108]. In another case report, despite treatment with antibiotics, antiviral, and antibacterial medications, a 64-year-old male COVID-19 patient developed respiratory failure on the 13th day of admission. The patient’s conditions improved following NAC supplementation, and discharge occurred after 46 days of hospitalization [109]. In particular, on the basis of its mucolytic and antioxidant properties, it has been proposed that inclusion of 1200 mg/d oral NAC in the therapeutic schemes of patients with COVID-19 could be an effective measure to prevent a cytokine storm and the associated acute respiratory distress syndrome [110].

A case-control study found that both H_2_S and NO were significantly higher in expired COVID-19 patients compared to those who survived, emphasizing the complex interaction between these two gasotransmitters and a more synergistic role in this context [111]. The reasons for this increase can hint a compensatory response of sicker patients to the detrimental effects of COVID-19 infection or, alternatively, an underutilization of NO and H_2_S, which results in fatal outcomes [111]. 

Finally, it has recently been proposed that another mechanism of defense against COVID-19 could involve the activation of the HO-1/CO/H_2_S system [112]. Indeed, HO-1 metabolizes the heme group of a variety of hemeproteins with the release of CO which, under endoplasmic reticulum stress conditions, inhibits CBS with decreased production of GSH, while the SH groups are enzymatically converted to H_2_S by CSE [112,113]. Liu and Li have hypothesized that ORF8 and the surface glycoprotein of SARS-CoV-2 attack and destroy the hemes of heme proteins, resulting in the suppression of HO-1 activation and H_2_S signaling [114,115]. Subjects with a long promoter for the HMOX1 gene (associated with decreased HO-1 anticoagulant activity) present an increased risk of recurrent venous thromboembolism [116], whilst HO-1 has been demonstrated to exert a significant antiviral activity against a wide variety of viruses [117]. Therefore, activation of the HO-1/CO/H_2_S axis has the potential to improve the clinical manifestations of COVID-19, and pharmacological treatments based on H_2_S delivery could once again represent an effective strategy in the treatment of patients with COVID-19 [112].

In addition to counteracting the inflammatory response in COVID-19, H_2_S may interfere with viral replication [96]. In fact, there is some well-established evidence demonstrating that H_2_S inhibits the replications of many other highly pathogenic RNA viruses in lungs [118], both by decreasing the expression of viral proteins and mRNA and by inhibiting syncytium formation and virus assembly/release [95,99].

Overall, a large array of data indicate that H_2_S could be a potential target for attenuating viral replication, inflammation development and progression, and organ damage, which needs to be further explored in preclinical models of viral infections [95].

## 3. Discussion

Amid the complexity of events and effectors underlying processes leading to endothelial dysfunction, H_2_S has emerged as one of the crucial determinants of endothelial homeostasis. Hence, sulfur drugs can represent advanced tools in the prevention and treatment of the numerous diseases involving endothelial dysfunction. For these reasons, several compounds are currently being investigated in clinical studies with promising results.

On the other hand, it has been recognized that a high H_2_S concentration (i.e., >250 ppm/~350 mg/m^3^) is harmful to health, and is associated with increased oxidative stress and inflammation [119]. Less well known are the long-term effects of chronic low-dose H_2_S exposures in light of controversial results and due to differences between and limitations of the available studies [119].

In addition to endogenous production, it is also important to consider the intake of H_2_S from exogenous sources. Indeed, H_2_S may originate from a variety of natural sources, including volcanoes, sulfur springs, undersea vents, swamps, bogs, crude oil and natural gas, or from man-made activities, such as oil refineries, tanneries, natural gas, products petrochemicals, ovens, food processing plants, municipal sewage and wastewater treatment plants, fertilization processes, and paper mills. Occupational exposure to H_2_S is therefore greater than that from environmental sources [119]. Furthermore, smokers have low serum H_2_S levels, while chronic alcohol users have high levels of H_2_S in their breath, suggesting that smoking and alcohol consumption may modulate endogenous H_2_S concentration [120].

Dietary intake can also affect endogenous levels of H_2_S. In addition to garlic, broccoli, mustard, etc., a high consumption of proteins and fats or a high carbohydrate content in the diet also appears to increase and reduce H_2_S levels, respectively [121]. In particular, the intake of sulfur in the diet, together with the presence and different composition of sulfate-reducing bacteria in the gastrointestinal tract, represent the most significant modulators of H_2_S production [121].

In this context, the discovery that H_2_S can protect the mucus layer and reduce inflammation when produced at nanomolar to low micromolar levels, exerting adverse effects when released at a higher concentration (from high micromolar to millimolar) by the local microbiota, is interesting because it has highlighted the double face of H_2_S in the balance between beneficial and harmful effects [122].

Although H_2_S donors may represent valuable tools to protect the endothelium, further clinical studies targeting their effect on the endothelium in terms of reducing or slowing the progression of dysfunction in specific diseases (e.g., COVID-19) would be desirable. Furthermore, it is currently unknown whether any drugs added to endogenous levels resulting from environmental exposure, lifestyle habits (cigarette smoking or alcohol consumption and diet) and microbiota activity could reach toxic concentrations, and this requires further refinement work towards a more personalized and targeted therapy for each patient.

## Figures and Tables

**Table 1:** Summary of the main biological effects of sulfur drugs.

Sulfur Drugs	Effects	Reference
**Inorganic sulfide salts** NaHS and Na2S	Reduction of inflammation, oxidative stress and damage induced by hyperglycemia; promotion of vasorelaxation and neovascularization	[6,52]
**Organic “slow-release” H_2_S compounds** GYY4137	Vasodilator, antihypertensive, anti-atherosclerotic, anti-thrombotic and anti-inflammatory effects	[38,41]
**Diphosphorothioates** AP67 and AP105	Promotion of high-glucose-induced hyperpolarization of the mitochondrial membrane and inhibition of ROS production in microvascular ECs,	[54]
**H_2_S prodrug sodium polysulthionate** (SG1002)	Promotion of increase in circulating H_2_S and NO and the consequent endothelial-dependent coronary artery vasorelaxation	[55,56]
**Natural organosulfur compound** Garlic Natural isothiocyanates	Promotion of vasorelaxation, lower arterial blood pressure, decreased apoptosis and oxidative stress, angiogenesis Anti-inflammatory and antioxidant effects	[57,58,59,60] [63,64,65]
N-acetyl-Cysteine (NAC) and taurine	Anti-hypertensive and anti-inflammatory effects	[66,67,68]
**Synthetic cysteine derivatives** (S-propyl-cysteine, S-allyl-cysteine and S-propargyl-cysteine)	Increase in H_2_S levels, anti-inflammatory effects	[69,70]
**H_2_S-hybrid drug** ACE inhibitors: Omaprilat, Remikiren, Macitentan, Bosentan, Vardenafil, Sildenafil	Pro-angiogenic, anti-inflammatory and anti-apoptotic activities Zofenopril: increase in H_2_S concentration in plasma and vascular wall	[71,72,73] [74]
**H_2_S-releasing derivatives of NSAIDs**	Anti-platelet, antithrombotic and antioxidant effects S-diclofenac (ACS15): anti-inflammatory and antiproliferative effects	[78,79,81] [82,83]

The classes of sulfur drugs are in bold.
